# Water-related fatalities in Salzburg and Upper Austria between 2015 and 2022 – a retrospective evaluation with a focus on the informative value of drowning signs

**DOI:** 10.1007/s00414-025-03675-z

**Published:** 2025-12-13

**Authors:** Dominik Hagen, Fabio C. Monticelli, Walter Stoiber, Peter Steinbacher, Stefan Pittner

**Affiliations:** 1 Dept. of Environment and Biodiversity, Paris-Lodron University of Salzburg, Salzburg, Austria; 2 Dept. of Forensic Medicine and Forensic Psychiatry, Paris-Lodron University of Salzburg, Salzburg, Austria

**Keywords:** Drowning, Retrospective analysis, Drowning signs, Decomposition

## Abstract

Drowning remains a major public health problem reportedly accounting for more than 4.000 annual deaths in the European Union. Factors influencing water-related fatalities are extending from intoxication through alcohol, pharmaceuticals and narcotic drugs to person-related medical conditions and the geography of the drowning environment. Consequently, cases suspected of drowning present a high variability in age, sex, seasonality, type of drowning, recovery site and state of body preservation by the time of recovery. All these factors affect the manifestation of drowning signs, which are investigated to enable a valid diagnosis of drowning. These drowning signs all result from physiological changes during the drowning process but are often non-specific in that they vary in abundance and severity of manifestation, also being prone to subsequent change by resuscitation efforts and decomposition processes, leaving the diagnosis of drowning as one of the most difficult tasks in forensic practice. By re-evaluating geographic, demographic, person-related and body condition data as well as diagnostic results of 158 water-related fatalities in Upper Austria and Salzburg from 2015 to 2022, this retrospective study reappraises the predictive strength of the drowning signs currently used in forensic practice, together with the influences acting on their formation and persistence. While some results are consistent with and affirm those of other studies with similar approaches, new relations were found regarding the differences in incidence rates between accident locations and water body types. Moreover, the main results identify that some drowning signs are of clearly higher predictive significance than others, and that predictive power increases with the number of signs coincidentally present but decreases with advancing decomposition. These findings underline the importance of a timely body recovery and in cases where not feasible, the need for additional alternative diagnostic measures.

**Supplementary Information:**

The online version contains supplementary material available at 10.1007/s00414-025-03675-z.

## Introduction

 The World Health Organization reports around 300.000 drowning deaths each year, listing drowning as the third leading cause of unintentional injury-related deaths worldwide and as a major public health issue [1]. The actual number of drowning cases is in fact likely to be significantly higher. Drowning events related to suicides, boat accidents or natural disasters are sometimes imprecisely investigated or reported, many victims never being discovered or otherwise not included in general drowning statistics [2]. Although most drowning cases originate from South-East Asia and the Western Pacific, drowning poses a major public health problem also in the EU, accounting for more than 4000 annual deaths and taking place wherever waterbodies are accessible [1–3]. General risk factors are inadequate swimming skills, overconfidence in own abilities and preexisting medical conditions [4–6]. While children are considered particularly at risk [7, 8], mostly as a consequence of a lack of supervision, young adults (especially men) also have a higher probability to drown due to higher risk taking and/or the consumption of alcohol [9–11]. Generally, most incidents occur in rivers or lakes and during warmer seasons but are also recorded throughout the entire year in various regions [12, 13]. However, not every corpse that is found in a body of water must necessarily have died by drowning. Therefore, even with further background information available, each case of suspected drowning requires specific actions of verification, especially close inspection by autopsy [14], to discriminate drowning from the immersion of persons already dead. Beyond the evaluation of classical drowning signs, each case must be inspected for patterns of trauma resulting from self-harm or third-party violence, pre- or postmortem sustained injuries, and other causes of death related to water-induced reflexes or underlying medical conditions, as seen in atypical drowning scenarios [14–16]. The typical drowning signs reflect pathophysiological alterations in various organ systems resulting from the drowning process. These signs include external foam cones extruding from mouth and nostrils, foamy liquid in the airways, emphysema aquosum (a specific type of pulmonary overinflation), Paltauf spots (hemorrhagic patches on the surface of the lower lung lobes), diluted/liquid stomach content, the Wydler sign (a three-layered pattern of gastric content sedimentation), the Svechnikov sign (free liquid in sphenoid sinuses), the Fritz sign (Sehrt sign) (longitudinal lacerations in the gastric mucosa) as well as intramuscular hemorrhages, splenic anemia and aortic hemolytic staining [17–21]. However, their specificity is limited as some signs may also be found in cases with other causes of death [22, 23]. Moreover, they may vary in abundance and severity of manifestation and can be masked by resuscitation efforts and decomposition processes [24, 25]. In addition, these signs may be only sparsely expressed or absent in cases of atypical drowning with a markedly shortened course [16]. Consequently, the diagnosis of drowning still represents one of the most difficult tasks in forensic practice and requires special caution in every respect [24, 26, 27].

To re-assess the factors known or thought to exert influence on drowning signs in more detail, we conducted a retrospective study comprising 158 water-related incidents from Upper Austria and Salzburg in the 8-year period from 2015 to 2022 and evaluated the impact of demographic affiliation, drowning site geography, season and body condition on the diagnostic output.

## Materials and methods

### Study design and data collection

This study was conducted on autopsy cases from an 8-year period (2015–2022) in the catchment area of the Institute of Forensic Medicine & Psychiatry Salzburg-Linz (which covers the Austrian federal states Upper Austria and Salzburg). Relevant fatalities associated with immersion or submersion in water were identified by screening autopsy records for keywords indicating a suspected drowning incident. Of 212 cases initially determined, 54 were excluded (1 duplicate, 2 with incompletely recovered bodies, 51 cases verifiably without relation to drowning, e.g. only scene location named after a nearby body of water), resulting in a final set of 158 cases. Relevant data records were then analyzed regarding their demographic, geographic and seasonal characteristics as well as autoptic findings, including the following parameters: type of water body, precise location of body discovery, date of occurrence, sex, age, manner of death and activity context, intoxication (alcohol, medication, illicit drugs), body condition (state of decomposition), presence of drowning signs and diagnosis on cause of death.

For detailed analysis, the following parameters were specified and/or grouped into subcategories: Locations of body discovery sites were associated with their postcodes to obtain more detailed information on the geographical cumulation of cases within the responsibility area. Body condition was partitioned into the three categories (i) fresh, (ii) early decomposition and (iii) advanced decomposition to examine seasonality and influence on the detectability of drowning signs and diagnoses. Cause of death diagnoses, which were primarily based on autopsy findings (sometimes supported by histology or diatom analysis), were classified into the categories (i) drowning, (ii) compatible-with-drowning, (iii) unclear and (iv) other cause of death.

Together, all relevant data were taken as a basis to identify the general risk factors for drowning and, as the principal aim, to reappraise the validity of drowning signs in the diagnosis of drowning, thereby also evaluating possible influences on these signs by attendant circumstances (e.g. resuscitation measures, season, putrefaction). However, it had to be considered with caution that the diagnostic classification had to be based on the very autoptic features that were subsequently subjected to statistical evaluation. Also, some additional indicators of drowning, such as aortic hemolytic staining, intramuscular hemorrhages and splenic anemia [19–21], were excluded from evaluation as they were not consistently reported.

### Statistics

χ^2^ tests were performed using Excel (Microsoft 365) and IBM SPSS Statistics 29 to evaluate intergroup differences and the relationship between variables and its categories (diagnosis of drowning, body condition, drowning signs), with values of *p* < 0.05 considered statistically significant. For multiple categories per variable, significance levels were adjusted by Bonferroni correction, and Fisher’s exact tests were used when the total numbers were less than five.

## Results

### Demographics, regionality and seasonal factors

**Fig. 1:**
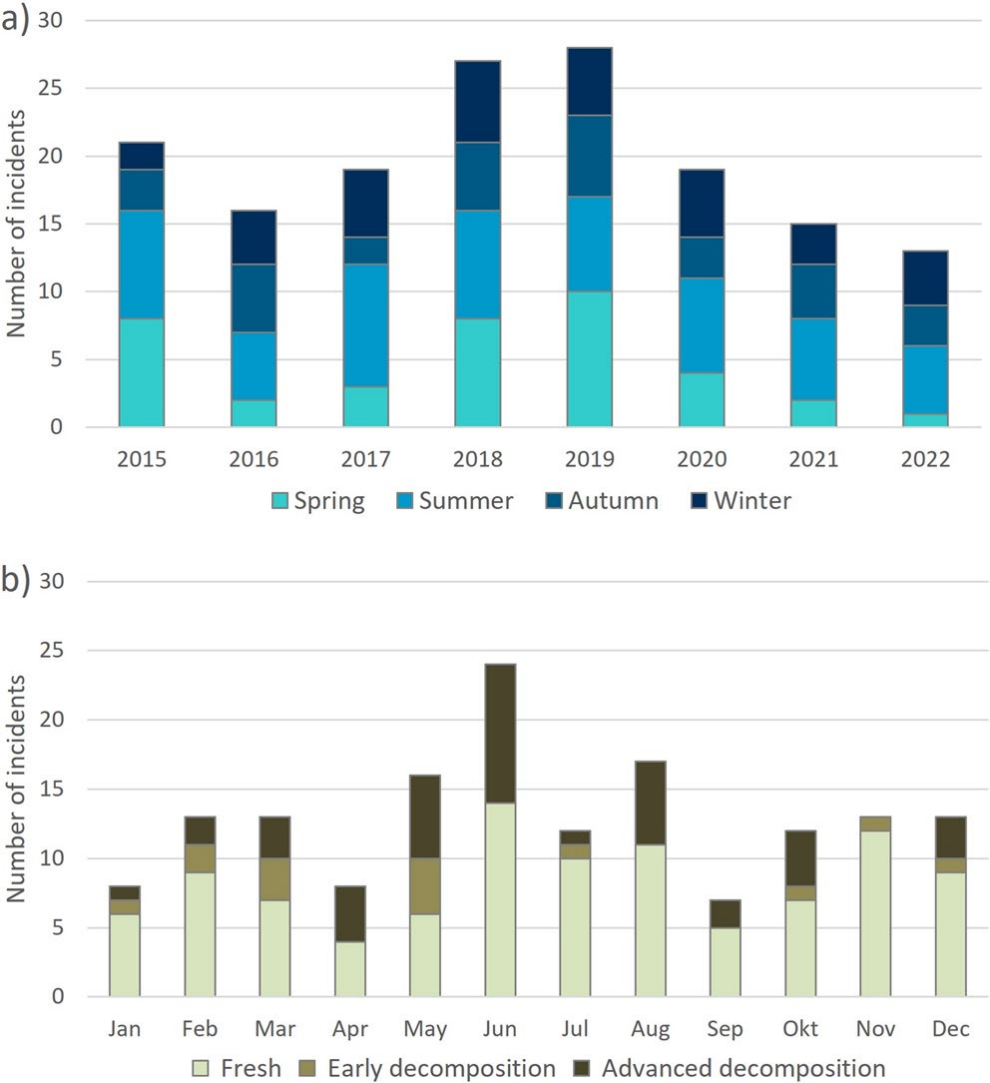
Water-related fatalities between 2015–2022, sorted by **(a)** Incidents per year and season, and **(b)** Incidents and respective body condition stages within individual months

This study included 158 water-related fatalities, comprising 103 male and 55 female cases with an average age of 52 years. Within the period investigated, the annual number of fatalities fluctuated considerably (Fig. 1a). The highest number of incidents were registered in 2018 and 2019, while lowest numbers were recorded in 2016, 2021 and 2022. Incidence also exhibited a characteristic pattern of seasonal variation (Fig. 1a + b). Especially in the years 2017, 2020, 2021 and 2022, most cases occurred during the warmer period from May to August, with an interim decrease in July. Incidence during all other seasons appeared more balanced, although with a trend to more cases in spring than in autumn and winter. This trend is particularly evident for the spring seasons of 2015, 2018 and 2019. By contrast, autumn and winter incidences fluctuated only slightly across months and between years, with lowest incidences in January, April and September. 100 cases of the 158 recovered bodies were not affected by decomposition at the time of discovery. Minor signs of decomposition were present in 14 cases, which were discovered during the colder period from October to March as well as in May and July. 44 cases were at advanced stages of body decomposition and predominantly, though not exclusively, discovered in the summer months.

All included cases were related to freshwater immersion. No drowning in other chemical fluids or liquid mixtures, as well as dry/asphyxial drownings were recorded.

Almost 85% of recorded fatalities occurred in natural bodies of water with an incidence of over 60% in rivers and streams, while standing waters such as lakes and ponds only accounted for 20%. The remaining cases occurred in domestic areas, mainly bathtubs and swimming pools.

**Fig. 2:**
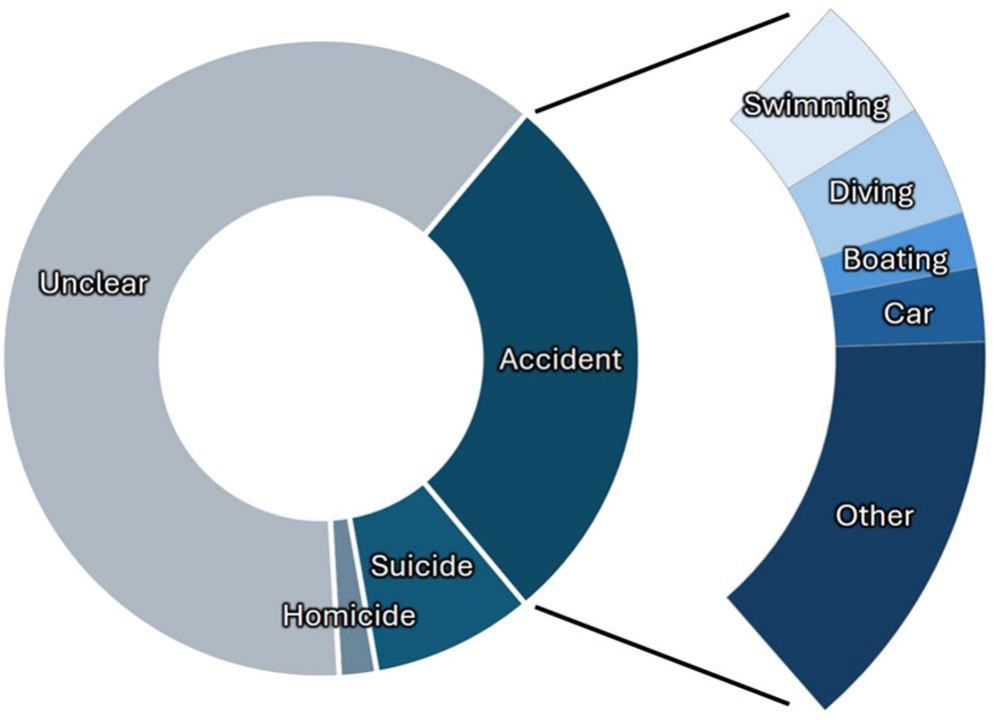
Manner of death in 158 water-related fatalities

In 27% of cases, the manner of death was related to accidents. More specifically, 10% were associated with water activities (swimming, diving, boating), 3% with accidents and 14% with other activities, such as mountain biking and hiking (Fig. 2). Apart from accidents, 8% of cases were suicides and 2% homicides, while in 63% of cases the manner of death was unknown or not specified.

Additional regional demographic and geographic information is provided in the supplements.

### Influence of detected drowning signs and body condition on the diagnosis

#### The diagnostic value of drowning signs

**Fig. 3:**
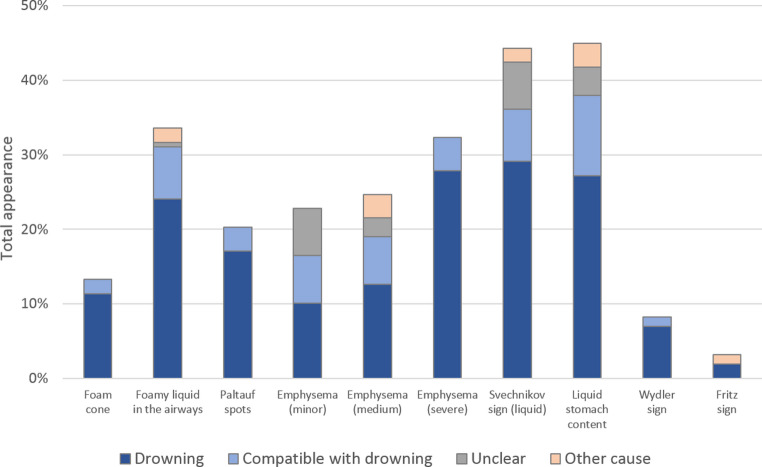
Total presence of drowning signs and allocation to diagnostic groups

The manifestation and mutual presence of drowning signs varied greatly between the re-examined cases (Table 1; Fig. 3). Although not considered a strong indicator of drowning, the most frequently detected sign was liquid stomach content. It occurred in 45% of all cases, notably in many where the cause of death was stated unclear or diagnosed as other than drowning. The Svechnikov sign (liquid in the sphenoid sinuses) was the second most frequently detected sign with 44%. It was also present in all diagnostic categories and observed in 14.3% of cases diagnosed as unclear and in 4.3% of cases with other cause of death, while approximately 60% of the cases lacking this sign were nevertheless associated with drowning. A more robust situation was found for the Wydler sign. It occurred in only 8% of cases but if present was always associated with a diagnosis of drowning. However, in 68% of cases a diagnosis of drowning was made in the absence of the Wydler sign. Similar significances were found for fully developed external foam cones and foamy liquid in the airways. Foam cones exuding from mouth and nostrils only appeared in 13% of cases but were always associated with drowning and never appeared in cases diagnosed as unclear and such of other cause of death. Foamy liquid in the airways was detected in 33% of cases and associated with drowning in 92%, although being also present in a few cases of unclear or other cause of death. However, 60% of drowning diagnoses were made in absence of this sign.

**Table 1:** Total presence of detected drowning signs and their relevance for the diagnosis (*p* < 0.05 is considered as statistically significant, n.s.= not significant)

Total appearance of drowning signs	Drowning	Compatible with drowning	Unclear	Other cause of death	*p*-value	odds ratio
**External foam cone**						
(+) (13%)	85.7%	14.3%	0.0%	0.0%	< 0.01	6.77
(-) (87%)	46.7%	19.7%	27.0%	6.6%		
**Foamy liquid in the airways**						
(+) (33%)	71.7%	20.8%	1.9%	5.7%	< 0.01	3.48
(-) (67%)	41.9%	18.1%	34.3%	5.7%		
**Emphysema aquosum**						
minor (23%)	44.4%	27.8%	27.8%	0.0%	n.s.	-
medium (25%)	51.3%	25.6%	10.3%	12.8%	n.s.	-
severe (32%)	86.3%	13.7%	0.0%	0.0%	< 0.01	11.22
(-) (20%)	6.3%	9.4%	71.9%	12.5%		
**Paltauf spots**						
(+) (20%)	84.4%	15.6%	0.0%	0.0%	< 0.01	6.89
(-) (80%)	43.7%	19.8%	29.4%	7.1%		
**Svechnikov sign**						
(+) (44%)	65.7%	15.7%	14.3%	4.3%	< 0.01	2.75
(-) (56%)	40.9%	21.6%	30.7%	6.8%		
**Liquid stomach content**						
(+) (45%)	60.6%	23.9%	8.5%	7.0%	n.s.	-
(-) (55%)	44.8%	14.9%	35.6%	4.6%		
**Wydler sign**						
(+) (8%)	84.6%	15.4%	0.0%	0.0%	< 0.05	5.68
(-) (92%)	49.0%	19.3%	25.5%	6.2%		
**Fritz sign** (Sehrt sign)						
(+) (3%)	60.0%	0.0%	0.0%	40.0%	n.s.	-
(-) (97%)	51.6%	19.6%	24.2%	4.6%		

High reliability as indicators for drowning could be assigned to the severe emphysema aquosum and the Paltauf spots. Although just detected in 32% and 20% of cases, respectively, both signs never appeared in any unclear case or a case of other cause. Severe emphysema proved particularly reliable, as only 16% of the drowning diagnosis but 72% of the decisions on unclear cause were made in its absence. By contrast, less pronounced stages of emphysema lacked such clear association with drowning and were more common in cases with unclear or other cause of death. With an overall occurrence of 3%, the Fritz sign was found to be the least frequent among the commonly monitored drowning signs. Its appearance was associated with a diagnosis of drowning in 60% of cases, while 40% of cases with this sign had a cause of death other than drowning. The absence of the Fritz sign (Sehrt sign) had no effect on the diagnosis of drowning in more than 70% of cases.

The revisited autopsy reports of 158 water-related incidents resulted in 82 cases (52%) diagnosed as ‘drowning’, 27 (17%) diagnosed as ‘compatible with drowning’, 9 (6%) with other causes of death such as polytrauma, barotrauma and respiratory failure and 40 cases (25%) which left the cause of death unclear.

**Fig. 4:**
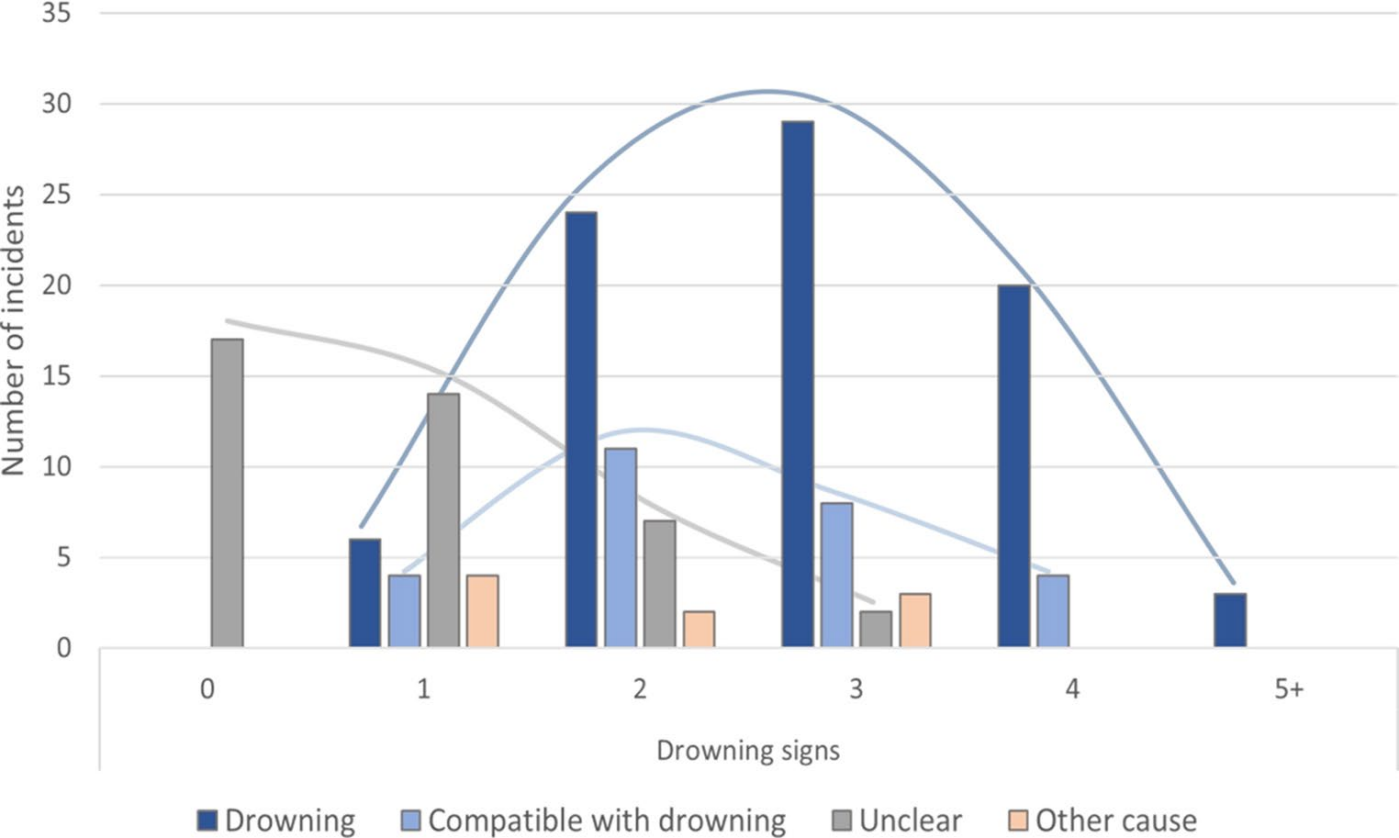
Number of detected drowning signs and respective diagnosis of all water-related incidents

In this regard, the present data demonstrates that the most robust basis for a decision is provided by the number of concurrently manifested drowning signs rather than the presence of any individual sign (Fig. 4). All cases with 5 or more simultaneously present drowning signs (*n* = 3 cases) were diagnosed as ‘drowning’. Similarly, approximately 83% of the 24 cases with 4 drowning signs were diagnosed as ‘drowning’ and 17% as ‘compatible with drowning’. These two diagnostic categories were also well represented among the cases with three (*n* = 42) and two drowning signs (*n* = 44) and limited in 10 individual cases with one drowning sign. When 3 drowning signs were present, 69% of cases were diagnosed as ‘drowning’ and 19% as ‘compatible with drowning’, while with 2 concurrently expressed signs, ‘drowning’ was diagnosed in 54% and ‘compatible with drowning’ in 25% of cases.

However, the present evaluation shows clearly that uncertainty about the diagnosis (category ‘unclear’) is inversely correlated with the number of present drowning signs. Accordingly, the percentage of unclear cases increased from 5% with three drowning signs to 16% with two drowning signs and 50% with 1 drowning signs. Cases without any sign of drowning were all diagnosed as ‘unclear’.

#### Impact of the body condition on drowning sign manifestation and diagnosis

**Fig. 5:**
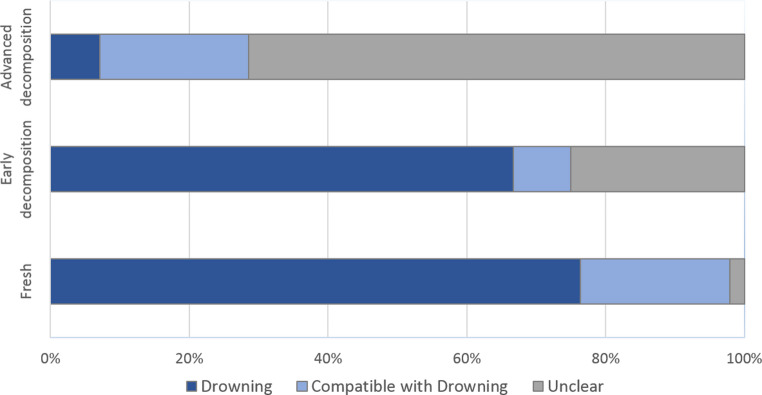
Relationship between the number of incidents (in %) and respective diagnosis as indicated by body condition

The state of decomposition by the time of body recovery (body condition) turned out to have a major influence on subsequent diagnosis (Fig. 5). Cases without any signs of decomposition (‘fresh’) were in 76% diagnosed as ‘drowning’ and in 22% diagnosed as ‘compatible with drowning’. By contrast, only 2% of such cases were assigned to the category ‘unclear cause of death’. However, these relations changed considerably when minor signs of decomposition were present. In such cases, the ratio diagnosed as ‘unclear’ increased to 25%, notwithstanding that a diagnosis of drowning was still possible in 75%. This trend became even clearer for cases in an advanced stage of decomposition, among which the ratio of cases diagnosed as ‘unclear’ rose to 72%, while the number of cases diagnosed as ‘compatible with drowning’ or ‘drowning’ dropped to 28%.

**Fig. 6:**
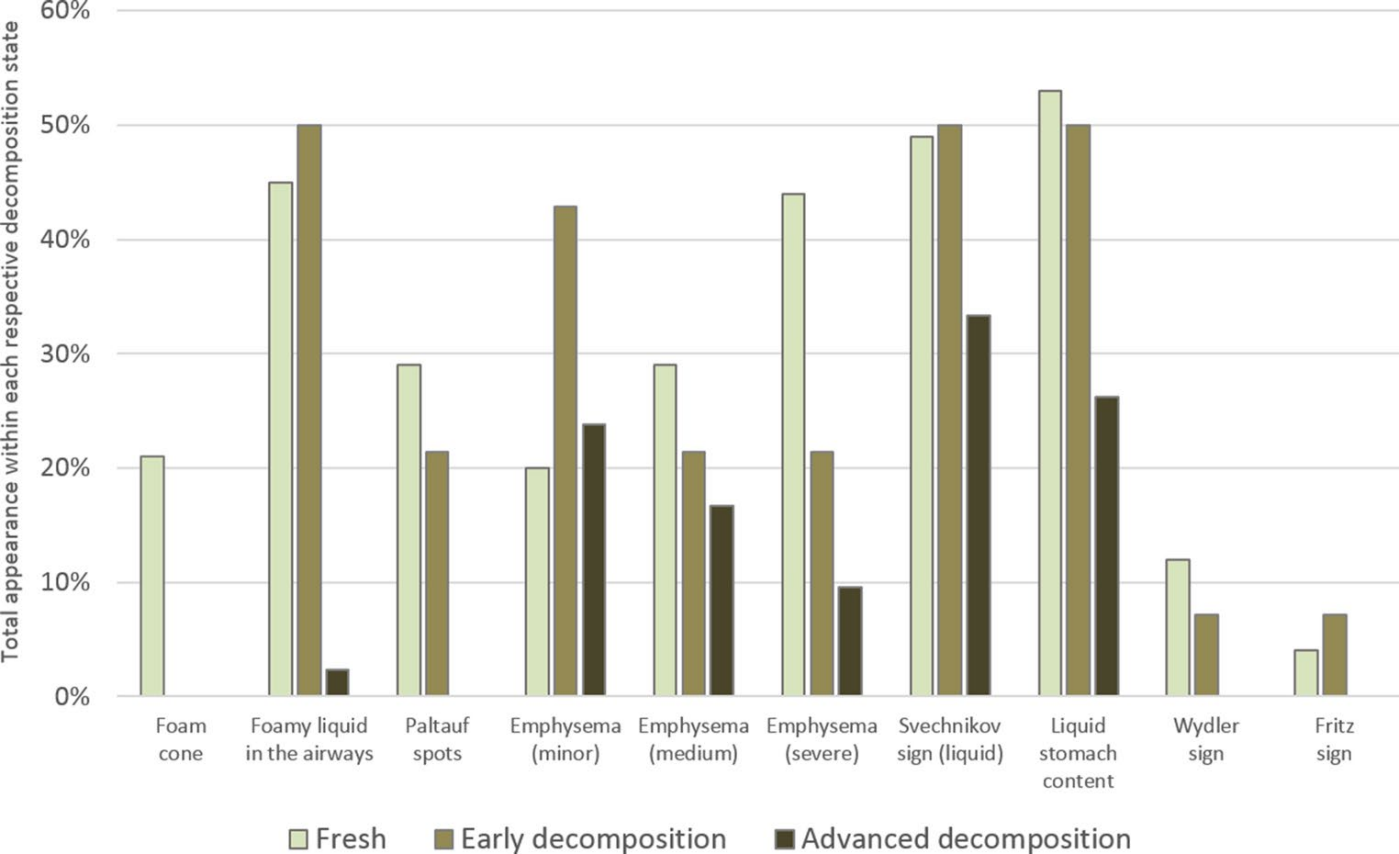
Relationship between the presence of individual drowning signs and respective body condition (Fresh = 100 cases, Early decomposition = 14 cases, Advanced decomposition = 44 cases)

The present data further reveal that manifestation and detectability of nearly all drowning signs changed considerably with the state of decomposition (Fig. 6). Thus, foam cones were confined exclusively to ‘fresh’ cases, while Paltauf spots, the Wydler sign and the Fritz sign were also observed in some cases of early decomposition state. Foamy liquid in the airways, the Svechnikov sign and liquid stomach content were found in all categories, although less frequent in cases within an advanced stage of decomposition. However, for the emphysema aquosum emerged a somewhat different situation. Minor emphysematous changes were detected more than twice as much in cases in a state of early decomposition than in the categories fresh and advanced decomposition, whereas numbers of cases with medium and severe emphysematous changes decreased with increasing decomposition.

### Toxicology

A total of 79 of the 158 subject cases (50%) were examined for antemortem consumption of alcohol, medication and/or illicit drugs prior to the incident. Of these cases, 33 were tested positive for alcohol, 16 for previous or ongoing medication, 9 for a mixed indication of both substances and 3 explicitly for illicit drugs (Table 2). Detected alcohol levels were considered potentially lethal in one case and as a contribution factor in 19 cases, while medication likely contributed in 10 further cases. In contrast, 18 cases were tested negative for all substances, of which 7 cases were considered negative despite slightly positive alcohol results as those were associated with advanced putrefaction processes.

**Table 2:** Toxicological analysis of alcohol, medication and illicit drugs in 79 water-related incidents

		Substances				
Sex	Tested	Alcohol	Medication*	Alc. & Med.*	Illicit drugs	Negative
m	56	24	9	6	3	14
f	23	9	7	3	0	4
Total	79	33	16	9	3	18

* Analgesics and psychotropic drugs

Although positive tests for substances or their mixed indications occurred more frequently in men than in women, the results appear quite balanced in terms of the number of cases per gender. 43% of the 56 tested men were found positive for alcohol, 16% for medication, 11% for mixed indication of both substances and 14% tested negative. Of the 23 tested women, 39% were found positive for alcohol, 30% for medication, 13% for both substances, and 17% tested negative. However, illicit drugs were exclusively detected among male incidents.

**Fig. 7:**
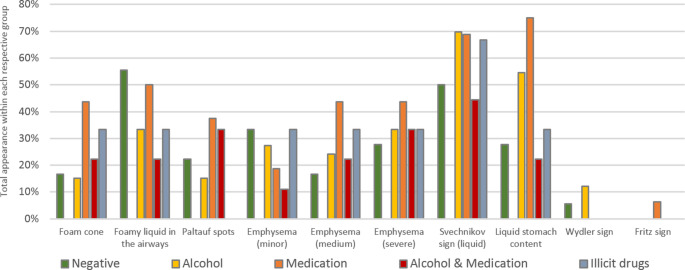
Impact of intoxicants on the manifestation of drowning signs

In addition, all intoxicants were tested for their impact on the presence of individual drowning signs. However, the results did not yet allow for clear conclusions, most likely due to case specific circumstances and sample size (Fig. 7).

## Discussion

This retrospective study presents several external factors attending water-related fatalities and re-evaluates their influence and value in the diagnosis of drowning incidents without ranking.

### Attendant factors and circumstances influencing the diagnosis of water-related fatalities

A conspicuous finding is the higher proportion of male victims, and in accordance with existing literature often attributed to increased risk-taking behavior and the underestimation of water hazards [9, 23, 28–32]. In many cases, prior accidents were responsible for subsequent drowning incidents and were related to recreational water activities in 10% of the cases. However, general contributing factors for water-related fatalities comprise reduced general fitness, overexertion, underwater currents, and pre-existing medical conditions [5, 6, 33, 34] as well as the underestimation of the dynamic flow behavior and the power of currents in running waters [35–37]. In fact, not lakes but rivers and streams proved to be predominant fatality locations in this study, presumably varying dependent on the given geographical conditions [10, 28, 38]. Annual and seasonal water-related fatality rates reveal an increased number of incidents during warmer periods of the years, consistent with previous research [11–13, 28].

These results also indicate indirect effects on diagnostic assessment. Factors such as seasonal temperature may alter the pace of microbial and molecular decomposition processes, likely affecting relevant diagnostic markers (see also 4.2 below) and the determination of the post mortal interval (PMI) [23]. Another considerable factor is river current, as it potentially complicates or impedes the localization of the incident site. Accordingly, it is quite possible that some incidents may have actually occurred in sidearms or tributaries and that the victims’ bodies subsequently drifted (ante- and/or postmortem) to the more or less distant discovery site [39]. In our study, such a scenario likely applied to at least 19 cases, which were all discovered at power plant dams of major rivers. Although analyses of diatoms or other microorganisms in water samples and victim’s tissues could provide insights about the incident site by comparing the species of different environments, it is often difficult to obtain reliable reference water samples and/or respective analyses aren’t commissioned [40–43].

Another important factor is toxicology and pharmacology. Particularly alcohol has been long known for its contribution to elevate drowning risk [24, 44–47]. This is confirmed by the present results, as almost 55% of the 79 tested cases were positive for alcohol and in half of them it was considered as contributing factor. Exclusive medication use was found in 19% of cases, whereas Illicit drugs (4%) only occurred rarely compared to other studies [5, 44, 48]. While illicit drugs and alcohol are known to impair judgement, motor coordination and reaction times, the impact of psychotropic drugs can only be speculated as the degree of impairment is difficult to determine by the given effects of such substances [5]. Nevertheless, a contribution was considered in 10 cases.

### The diagnostic reliability of drowning signs

The diagnosis of drowning remains a particular challenge in forensic practice, relying on investigative information, the exclusion of other causes of death and the detection of several macroscopic autoptic findings associated with drowning [14]. The results of this retrospective analysis contribute to a better understanding of the predictive power of the individual drowning signs and their interference with masking effects resulting from concurrent influences including postmortem decomposition. Although none of these signs are highly specific or definitively indicative for drowning, they are still essential for diagnosis [49]. Some signs appeared to be more predictive and relevant for the diagnosis than others, due to their frequency of occurrence, the influence by masking factors or postmortem decomposition processes, as well as their concurrent expression.

In accordance with other studies [50–52], the most frequently found drowning sign (80%) were emphysematous changes of the lung. However, their predictive power turned out to depend on the intensity of expression. Only a severe emphysema aquosum provided strong support to the drowning diagnosis, while minor and medium emphysematous changes were found in all diagnostic categories. Nevertheless, also the latter may have a minor indicative effect, since only about 16% of the cases without any emphysema could be reconciled with drowning.

A group of other drowning signs (albeit less frequently observed) also disclosed strong predictive strength. One of these signs is Paltauf spots, which were found in 20% of cases and always coincided with a drowning diagnosis. This high validity and significance is also in agreement with the available literature, regardless of the highly varying expression rates (10.6%−70%) [49, 51]. This evidential value also applied for external foam cones (present study 13%, literature 4%−17%) [50–53] and, with somewhat lower validity, for foamy liquid in the airways (present study 33%, literature 28%−46%) [54, 55]. This group of drowning signs also includes the by far less common Wydler sign. Although only present in 8% of cases in our study (literature 13%−16%) [51, 56], it was always associated to a diagnosis of drowning.

By contrast, the remaining three signs turned out to be less conclusive with little predictive strength as being present in all diagnostic categories. This applied to liquid stomach content (45%, expression rate within the known range) [23, 51, 52, 56, 57], the Svechnikov sign (44%, with partial support from literature [51, 52, 58–60] and to the rarely occurring Fritz sign (3%, similar to other studies) [51, 61].

However, when using drowning signs in drowning diagnosis, it must be considered that their expression is individually susceptible to various secondary influences and can be harshly limited in cases of atypical drowning. Other than the drowning process itself additional factors may significantly modify drowning signs. These include aspects of the aquatic environment, such as osmolarity, currents or tidal effects, or injuries caused by drift and animal predation [24, 62, 63], but also resuscitation measures and most prominently decomposition.

Of all drowning signs investigated in this study, the emphysema aquosum appeared as the most promising sign but turned out to be highly susceptible to external factors. When comparing the expression rate with body condition, minor emphysematous changes were more frequently observed in early and advanced decomposition cases. However, its predictive value is limited precisely by the fact that minor emphysematous changes of the lung can be induced not only by resuscitation measures or decomposition-related gas formation, but can also occur as a consequence of pre-mortal aging and disease and thus be detected even in non-drowning cases [15, 26, 51, 64, 65]. By contrast, the external foam cone, even though being highly indicative when present, is reportedly only detectable within the first 24 h after the actual incident and tends to vanish with prolonged PMI or upon the initiation of resuscitation measures [53, 66, 67]. Foamy airway liquid may persist for a somewhat longer period [51], but its validity in drowning diagnosis decreases in case of drug intoxication and may also be observed in cases of postmortem submersion [14, 24, 54, 63]. While the Fritz sign is prone to false positive elicitation by resuscitation measures [22, 61], Paltauf spots and the Wydler sign appear largely immune to such measures [51]. However, they all yield the disadvantage of potential absence in advanced decomposition cases. Additionally, the identification of Paltauf spots and Wydler sign requires special caution. Paltauf spots are at risk to be confused with the very similar Tardieu spots [51], while the Wydler sign is generally subject to the individual interpretation of the autopsy specialist, as the gastric sediment layers to be measured may vary considerably in volume. A slightly different situation limits the predictive power of the Svechnikov sign. Although not affected by resuscitation measures and detectable at all stages of decomposition, its significance in the diagnosis of drowning is attenuated by the circumstance that liquid amounts in the sinuses may be increased by the accumulation of decomposition fluid [51].

The influence of alcohol, medication or other intoxicants on the manifestation and persistence of any individual drowning sign could neither be confirmed nor denied and would certainly require a broader database to provide valuable results. Since half of the 158 re-examined cases were lacking specific commissioning, it would be advisable to adapt the regulatory procedures to ensure that all water-related incidents are subject to toxicological screening.

To conclude, none of the drowning signs known to date fully meets ideal specifications, as nearly all can be affected by pre- and/or postmortem influences. Nevertheless, the present results show that some signs should be preferred regarding their predictive strength for the diagnosis of drowning. These signs include the severe emphysema aquosum, external foam cones, the resuscitation-unaffected Paltauf spots and Wydler sign, as well as to a somewhat limited extent also foamy liquid in the airways and the Svechnikov sign. The remaining signs (medium/minor emphysema aquosum, liquid stomach content and the Fritz sign) must be classified as clearly less significant.

However, the highest levels of predictive strength are always achieved when several strong drowning signs are simultaneously present. Only a very few cases with two or three of these signs being detected were attributed to causes of death other than drowning or rated as unclear due to excessive decomposition. Cases with four or more of these signs were always reconciled with a drowning incident. Nevertheless, it must be added that the presence of many drowning signs is not mandatory for a drowning diagnosis. A few cases exhibited only a single drowning sign but were still associated with drowning, since the respective identified drowning sign was of high validity and intensely expressed, or because additional investigative information about the incident, such as eyewitness reports, could contribute to the diagnosis [14]. By contrast, all cases diagnosed as unclear were in a state of advanced decomposition and either showed no signs of drowning or of low intensity or validity and were consequently inconclusive.

In summary, the findings of this study further attribute the epidemiological and diagnostical complexity of this public health issue and identify possible starting points for targeted prevention strategies to improve education for respective risk groups and (in line with the motto ‘don’t drink and dive’) restrict alcohol access for water activities [4, 5, 68]. Despite the relevance of such preventive approaches, attention must still be paid to the diagnostic assessment itself. Even if some drowning signs appear more valid than others, their combined presence remains indispensable for final diagnosis. The negative influence of decomposition processes on the expression of drowning signs and thus on the diagnosis further underpins that discovering a victim’s body in due time is of considerable importance. With bodies found late after the drowning incident, it could be helpful to envisage additional investigatory measures [69, 70]. Concerning this, regular histological examinations and microbiological comparisons of water bacteria [71, 72] or diatoms recovered from the victims tissue and presumed drowning locations could provide additional evidence of alive water contact and a subsequent death by drowning [40, 43, 73–75].

.

## Supplementary Information

Below is the link to the electronic supplementary material.

Supplementary Material 1 (PDF 312 KB)

## Data Availability

All data generated or analyzed in course of this study are included in the article.
