# Supplementary material for: Water-related fatalities in Salzburg and Upper Austria between 2015 and 2022 – a retrospective evaluation with a focus on the informative value of drowning signs
Source: Int J Legal Med. 2025 Dec 13;140(2):825–36. doi: 10.1007/s00414-025-03675-z (PMC12956979; doi:10.1007/s00414-025-03675-z)
Supplement: Supplementary file 1 — Supplementary Material 1 (PDF 312 KB) [file 414_2025_3675_MOESM1_ESM.pdf]

Supplements

Regional demographic and geographic data

The age distribution of water-related fatalities showed the lowest numbers in the 0–15 years age class, with no difference between males and females (3 cases each) (Fig. S1). The 16-30 years and 31–45 years age classes showed an increase to 19 and 27 fatalities respectively, each with a three times higher death rate in men. The highest number of fatalities (44) was recorded in the 45-60 years age class, the second highest (35) in the 60-75 years class, while in both classes the number of females was only slightly smaller than the number of males. This balanced sex ratio is also in agreement with established knowledge [76]. By contrast, the 75+ age class, with a total of 24 fatalities, is again characterized by a clear overhang of men. The higher risk of drowning in age groups 46-75+ has been attributed to age-related physical decline and underlying medical conditions [33,77,78]. This stands in clear contrast to incidents among younger adults which are predominantly associated with increased risk-taking behavior, the overestimation of physical abilities and underestimation of water-related dangers [9].

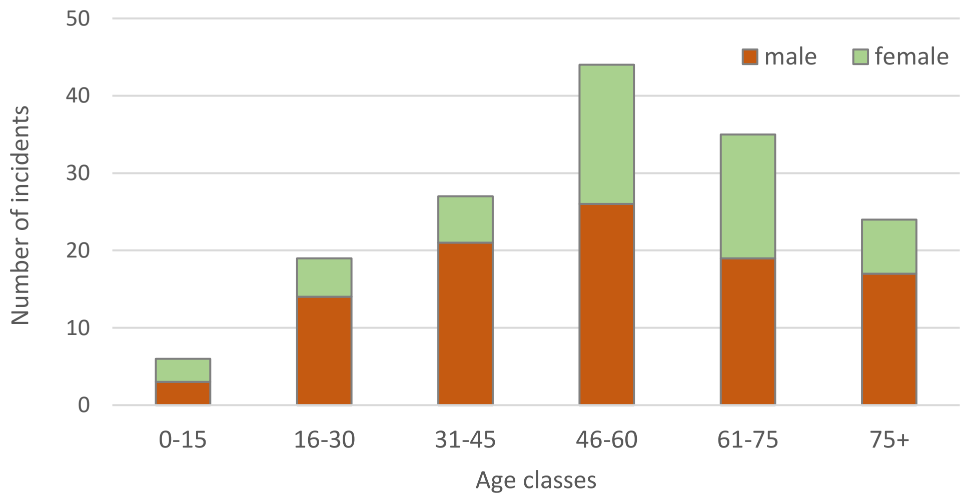

Fig. S1 Water-related fatalities in the period of 2015-2022, sorted by age class and sex

Highest incidences of water-related fatalities in Upper Austria were registered for the rivers Danube, Traun and Inn, and in Salzburg for the Salzach river and its tributaries such as the Mühlbach stream. The highest rate in standing waters was recorded for the Attersee, Austria's largest inland lake (46,2 km^2) bordered by both federal states (Table S1, Fig. S2).

Postal code-based analysis of recovery sites (Fig. S2) reveals that around 60% of all fatalities were located in Upper Austria, a state with 1.53 million inhabitants allocated to 400 postal

codes in a territory of 11.982 km². 40% of the fatalities were located in the clearly smaller and less populated state of Salzburg (560.000 inhabitants, 130 postal codes in a territory of 7.053 km²). In both states, more densely populated areas showed higher incidences than less populated regions, but fatalities occurred wherever there was access to any kind of public or private water body. Geographically, cumulation sites of fatalities were identified specifically in waters around larger cities with over 50,000 inhabitants [28]. However, in contrast to some other studies and likely varying dependent on the given geographical conditions [10,28,38], not lakes but rivers and streams proved to be predominant locations, accounting for 49% and 16% of all incidents in this study, whereas incidents in lakes only accounted for a total of 14%. Notably, all incidents in lake Attersee were diving accidents, most likely related to specific (scenic) sediment patterns in the diving area at the east bank. These sediment patterns are believed to induce deceptive perception of water depth, leading divers to misjudge the lake's actual water level and resulting in fatal accidents when oxygen is no longer sufficient to reach the surface safely (three atypical drownings, two typical drownings, one unclear case).

Table S1 Distribution of water-related fatalities according to type of water body and site of recovery

Body of water	Name	Drowning cases	Total cases (in %)
**river**	Danube	31	77 (49%)
	Traun	11	
	Salzach	8	
	Inn	7	
	other	20	
**stream**	Mühlbach	4	26 (16%)
	other	22	
**lake**	Attersee	6	22 (14%)
	Traunsee	4	
	other	12	
**pond**		10	10 (6%)
**domestic**	bathtub	8	8 (5%)
	other	15	15 (9%)
**total**		158	158 (100%)

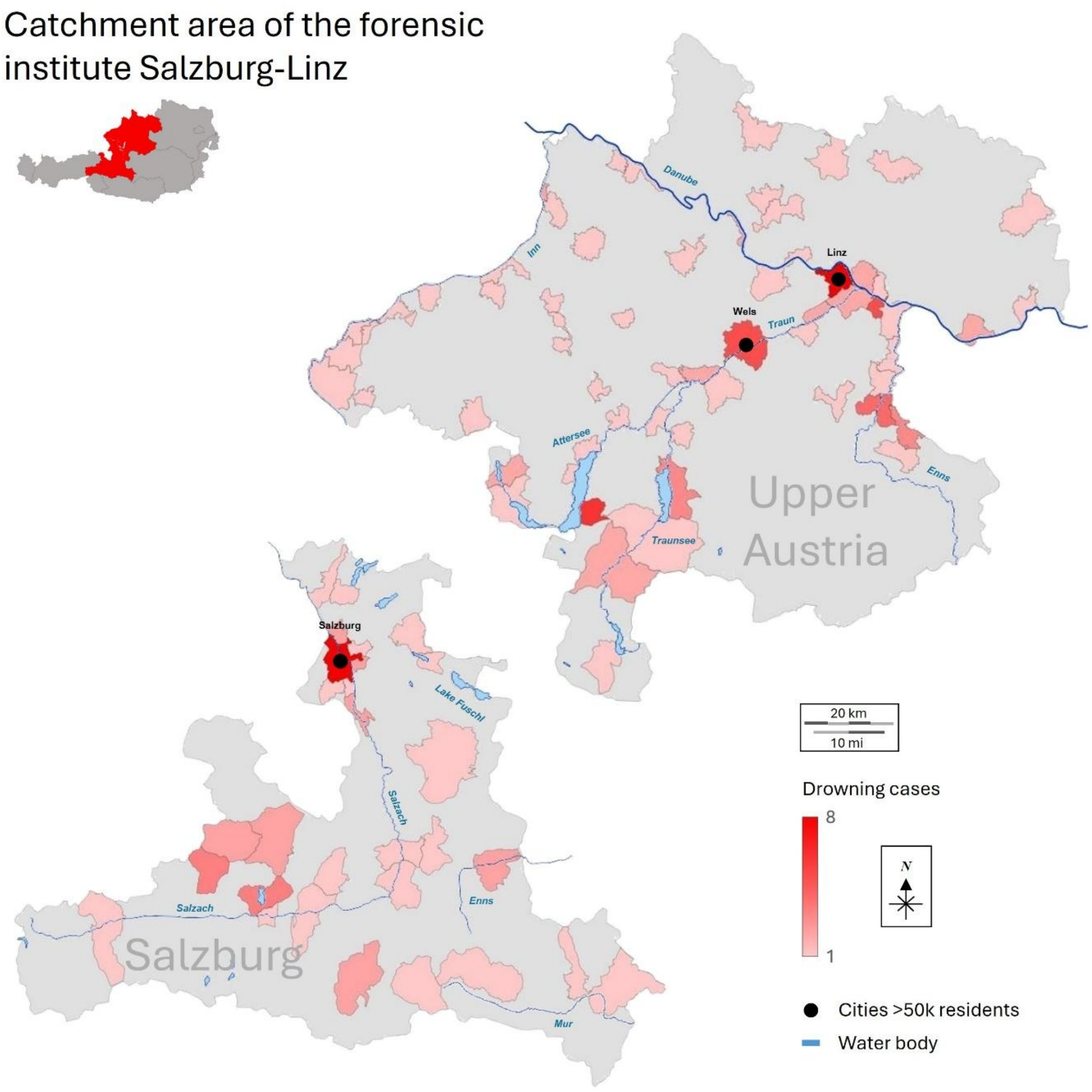

Fig. S2 Silent postal code-based distribution map of water-related fatalities in Upper Austria and Salzburg. (Smaller rivers, streams, lakes and ponds not shown).
